# *QuickStats:* Percentage Distribution of Deaths Involving Injuries from Recreational and Nonrecreational Use of Watercraft,* by Month — United States, 2015–2017

**DOI:** 10.15585/mmwr.mm6828a4

**Published:** 2019-07-19

**Figure:**
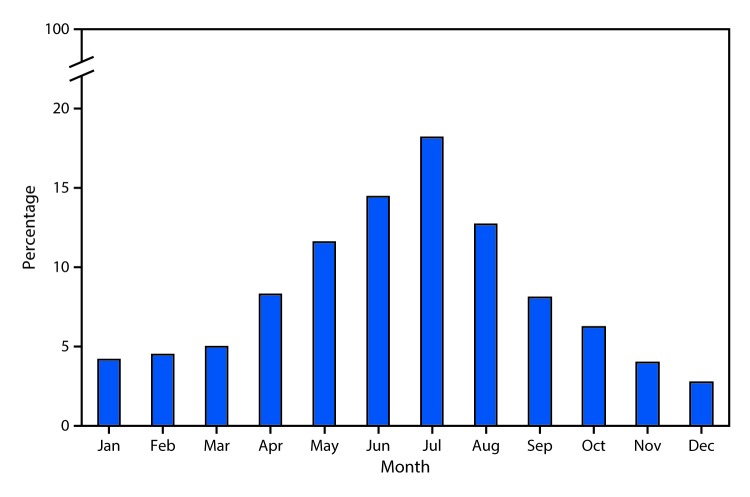
During 2015–2017, there were 1,389 deaths involving injuries from recreational and nonrecreational use of watercraft (an average of 463 deaths per year). The percentage of deaths that occurred by month ranged from 2.7% in December to 18.2% in July. The majority of deaths (57%) occurred during May–August.

**Source:** National Center for Health Statistics, National Vital Statistics System, Mortality File. https://www.cdc.gov/nchs/nvss/deaths.htm.

